# Benefit of Ambulatory Management of Patients with Chronic Heart Failure by Protocolized Follow-Up Therapeutic Education and Remote Monitoring Solution: An Original Study in 159 Patients

**DOI:** 10.3390/jcm9103106

**Published:** 2020-09-25

**Authors:** Anne Jenneve, Noel Lorenzo-Villalba, Guy Courdier, Samy Talha, François Séverac, Abrar-Ahmad Zulfiqar, Patrick Arnold, Philippe Lang, Gérald Roul, Emmanuel Andrès

**Affiliations:** 1 Unité de Suivi des Patients Insuffisants Cardiaques, Clinique du Diaconat, 68067 Mulhouse, France; anne@jenneve.net (A.J.); guycour68@gmail.com (G.C.); patrick.arnold@gmail.com (P.A.); philippe.lang@gmail.com (P.L.); 2 Service de Médecine Interne, Diabète et Maladies Métaboliques, Clinique Médicale B, Hôpitaux Universitaires de Strasbourg, 67000 Strasbourg, France; noellorenzo@gmail.com (N.L.-V.); abzulfiqar@gmail.com (A.-A.Z.); 3 Equipe de Recherche EA 3072-Mitochondrie, Stress Oxydant et Protection Musculaire, Faculté de Médecine de Strasbourg, Université de Strasbourg, 67000 Strasbourg, France; samy.talha@chru-strasbourg.fr; 4 Service de Physiologie et d’Explorations Fonctionnelles, Hôpitaux Universitaires de Strasbourg, 67000 Strasbourg, France; 5 Département de Santé Publique/DIM et Biostatistiques, Hôpitaux Universitaires de Strasbourg, 67000 Strasbourg, France; francois.severac@chru-strasbourg.fr; 6 Unité Fonctionnelle Dédiée à L’insuffisance Cardiaque, Pôle Médical et Chirurgical des Maladies Cardio-vasculaires, Hôpitaux Universitaires de Strasbourg, 67000 Strasbourg, France; gerald.roul@chru-strasbourg.fr

**Keywords:** heart failure, protocolized follow-up, therapeutic education, remote monitoring solution, telemedicine, care pathway, coordination unit, personalized medicine, telemedicine

## Abstract

This study sought to determine whether the implementation of regular and structured follow-up of patients with chronic heart failure (CHF), combined with therapeutic education and remote monitoring solution, leads to better management. This was a single-center retrospective study conducted in a cohort of patients with proven CHF who were followed up in the Mulhouse region (France) between January 2016 and December 2017 by the Unité de Suivi des Patients Insuffisants Cardiaques (USICAR) unit. These patients received regular protocolized follow-up, a therapeutic education program, and several used a telemedicine platform for a two-year period. The primary endpoint was the number of days hospitalized for heart failure (HF) per patient per year. The main secondary endpoints included the number of days hospitalized for a heart condition other than HF and the number of hospital stays for HF per patient. These endpoints were collected during the year preceding enrollment, at one year of follow-up, and at two years of follow-up. The remote monitoring solution was evaluated on the same criterion. Overall, 159 patients with a mean age of 72.9 years were included in this study. They all had CHF, mainly NYHA Class I-II (88.7%), predominantly of ischemic origin (50.9%), and with altered left ventricular ejection fraction in 69.2% of cases. The mean number of days hospitalized for HF per patient per year was 8.33 (6.84–10.13) in the year preceding enrollment, 2.6 (1.51–4.47) at one year of follow-up, and 2.82 at two years of follow-up (1.30–6.11) (*p* < 0.01 for both comparisons). The mean number of days hospitalized for a heart condition other than HF was 1.73 (1.16–2.6), 1.81 (1.04–3.16), and 1.32 (0.57–3.08), respectively (*p* = ns). The percentage of hospitalization for HF for each patient was 69.5% (60.2–77.4), 16.2% (10–25.2), and 19.3% (11–31.8), respectively (*p* < 0.001 for both comparisons). In the group telemedicine, the mean number of days hospitalized for HF per patient per year was 8.33 during the year preceding enrollment, 2.3 during the first year of follow-up, and 1.7 during the second. This difference was significant (*p* < 0.001). The “number of days hospitalized for a heart condition other than HF” was significantly reduced in the group of patient’s beneficiating from the remote monitoring solution. This study demonstrates the value of a protocolized follow-up associated with a therapeutic optimization, therapeutic education program, and the use of a remote monitoring solution to improve the management of ambulatory patients with CHF, particularly of moderate severity.

## 1. Introduction

According to a 2017 report by the *Institut de Veille Sanitaire*, the French public health surveillance institute, the estimated prevalence of chronic heart failure (CHF) among adults in France is 2.3%, a figure that represents more than 1 million inhabitants [1,2]. Heart failure (HF) is the main cause of hospitalization among patients aged 65 and over [1]. Since 2010, it has been the third most common cause of death among adults under 65 in France, after cancer and violent deaths [3]. HF treatment has progressed considerably in recent years, leading to a noticeable improvement in prognosis. This is true of pharmacological treatments such as combinations of angiotensin II receptor blockers and neprilysin inhibitors (valsartan/sacubitril) and gliflozins like empagliflozin and dapagliflozin [4,5]. Also effective are some non-pharmacological treatments, particularly the patient taking ownership of the disease, following dietary and lifestyle guidelines, and tackling sedentary behavior through a patient education program and the establishment of a clinical pathway for the follow-up of patients with CHF [6,7]. In this setting, telemedicine may be of significant help, given that it can be instrumental in optimizing CHF management, particularly by preventing emergencies such as acute HF and repeated iterative hospitalizations [6]. Likewise, telemedicine may help make it possible to better structure integrated care pathways [6,7].

Our aim was to establish whether implementing a regular, structured follow-up for patients with CHF combined with a patient education program and a remote monitoring solution led to better patient management.

## 2. Patients and Methods

### 2.1. Aim

Our study investigated the effect of a regular, systematized follow-up, a patient education program, and a remote monitoring solution on the prognosis of patients with CHF, where both the follow-up and education program were run by a coordinating unit.

### 2.2. Method

This retrospective single-center study was conducted in a cohort of patients with confirmed CHF who were treated in the Mulhouse region of France between January 2016 and December 2017 at the *Unité de Suivi des Patients Insuffisants Cardiaques* (USICAR) HF unit. Eligible patients were identified following hospitalization for HF at the *Fondation de la Maison du Diaconat de Mulhouse*, a group of private non-profit care centers in Mulhouse (France).

During the follow-up, all the included patients have benefited from a protocolized follow-up and a therapeutic education. A telemonitoring solution (*TransData System*, Mulhouse, France) was also proposed to all included patients likely to use it.

### 2.3. Inclusion and Exclusion Criteria

The patients enrolled were adults (≥18 years) with CHF who gave their consent to take part in the follow-up and education program developed by the USICAR HF unit. The diagnosis of CHF was made using clinical, laboratory, and ultrasonographic criteria according to the criteria of the *European Society of Cardiology* (ESC) [7]. All patients hospitalized at the *Fondation de la Maison du Diaconat de Mulhouse* for cardiac decompensation were potentially eligible for enrollment in our study. However, only those patients who had retained their physical and mental capacities—those who were mobile and could understand—were considered for enrollment in the USICAR program.

Children and pregnant women were excluded, as were subjects who were incapable of expressing their opinion or who were at risk of dying within 2 months from any cause.

### 2.4. Study Endpoints and Data Collection

#### 2.4.1. Primary Endpoint

The primary endpoint of the study was the number of days hospitalized for HF per patient per year. This endpoint was measured during the year preceding enrollment, after 1 year of follow-up in the USICAR program, and after 2 years of follow-up.

#### 2.4.2. Secondary Endpoints

Our secondary endpoints were the number of days hospitalized for a heart condition other than HF (including acute coronary syndrome, pacemaker placement, and correction of hypertension), and the number of hospital stays for HF per patient. These two endpoints were also measured during the year preceding enrollment, after 1 year of follow-up in the USICAR HF unit, and after 2 years of follow-up. Patient knowledge was assessed first at enrollment and then when patients left the USICAR program by means of a questionnaire testing the patient’s knowledge. The improvement of the treatment was also studied in terms of drugs and doses with respect to the standard treatment of CHF patients according to the ESC recommendations [6,7]. Additionally, the effect of the follow-up and education program offered by the HF unit on overall mortality and care costs was investigated during the year preceding treatment at the unit and after 1 and 2 years of follow-up. For the purposes of this cost analysis, only the cost of hospitalization for HF was considered. The mean total for such treatment was €4700, with the money spent on running the USICAR HF unit (mean of €600 per patient per year) included.

An analysis according to the same criteria (number of days hospitalized for HF per patient per year, number of days hospitalized for a heart condition other than HF) was also carried out for the group of patients benefiting from a remote monitoring solution.

#### 2.4.3. Data Collection

All data were collected by the care team during follow-up visits, entered into the patients’ medical records, and consolidated after the study by telephoning family physicians, community cardiologists, and hospital departments. The effect of the education program was assessed using a HF knowledge questionnaire which was administered to the patients at the beginning of each visit.

### 2.5. USICAR HF Unit

**Figure 1:**
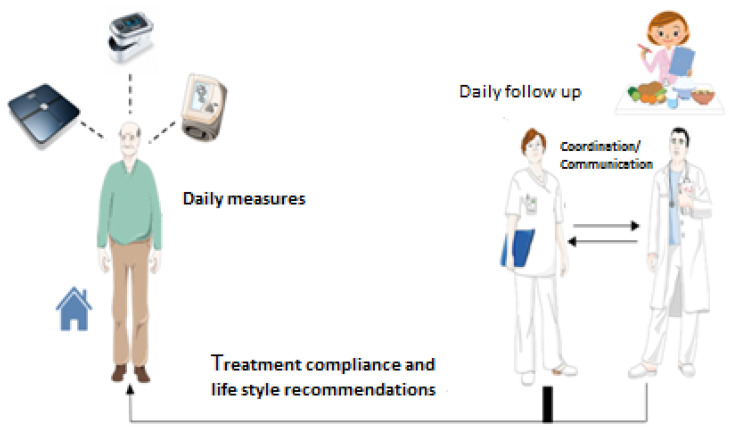
The USICAR (*Unité de Suivi des Patients Insuffisants Cardiaques*) HF (Heart Failure) unit is aimed at optimizing the management of HF patients, especially those recently hospitalized for cardiac decompensation. The team of the unit is composed of three cardiologists, two nurses, one dietitian, and one secretary. The unit ensures a regular personalized follow-up and therapeutic education of heart failure patients, as well as the coordination with the other healthcare professionals involved in the management of each patient. A remote monitoring solution was used in all patients likely to use it.

The USICAR HF unit is a treatment center specifically for patients with CHF located in the Mulhouse region of France. It coordinates a hospital–community care network that follows up patients with CHF, usually after they have been discharged from hospital. The unit oversees coordination between the hospital and external care providers such as family physicians, hospital or community cardiologists, and home care nurses. It provides various services to ensure continuous, optimal patient management, including through home patient monitoring and patient education. The USICAR HF unit is organized along the lines of a care and clinical pathway coordination unit (Figure 1).

The USICAR HF unit is aimed at optimizing the management of HF patients, especially those recently hospitalized for cardiac decompensation. In this setting, the objectives of this unit are: Follow-up and analysis of the daily measurements (blood pressure, cardiac frequency, and weight); weekly follow-up of the patient (symptoms and signs as shortness of breath, weight gain, edema, fatigue); compliance with treatment and dietary-lifestyle recommendations; treatment adaptation; coordination and communication with all healthcare professionals; and, last education of the patient with the USICAR program. In this setting, a remote monitoring solution (including a website and a personal scale) was proposed in patients able to use it (this solution was available six months after the beginning of the project).

### 2.6. USICAR Program

The USICAR program was developed and launched by the cardiologist Dr. G. Courdier and conducted in accordance with, and with the support of, the *Agence Régionale de Santé d’Alsace* (Alsace Regional Health Agency). In this program, all patients with CHF received an initial one-on-one consultation with one of the cardiologists of the team. This cardiologist was accompanied by a nurse and a dietitian trained in HF management who carried out personalized educational and dietary assessments on the patients. Thereafter, the patients saw these practitioners at regular visits at least once every three months to ensure that follow-up and patient education complied with good clinical practice and the guidelines of the *Haute Autorité de la Santé* (French National Authority for Health) for the management of patients with CHF [6]. Between these visits, the patients were regularly telephoned by the nurses. The frequency of these calls was defined with each patient according to a personal care plan drawn up with them in the beginning, but at least once a week. During these telephone calls, the patients and nurses discussed the patients’ most recent blood pressure and weight readings, the presence or absence of dyspnea and edema of the lower extremities, and adherence to treatment, diet, and lifestyle. Particular attention was given to the triggers of cardiac decompensation most commonly reported by the French National Authority for Health. These triggers are non-adherence to treatment and non-compliance with prescribed dietary and lifestyle behaviors [6].

### 2.7. Statistical Analysis

Continuous variables were presented as mean, range, and percentage. Normality was assessed graphically and using the Shapiro–Wilk test. Categorical variables were described as number and percentage. “Number of days hospitalized” being a countable variable, this endpoint was compared between the follow-up periods using a Poisson regression model. Given that these were repeated measures comparing the same subjects at different times, a generalized estimating equations (GEE) approach was used with an unstructured correlation matrix to take account of intra-subject correlations. The duration of observation of the patients was entered into the model as an offset to compensate for differences between follow-up periods due to deaths or patients lost to follow-up. The results were presented as ratios of means and 95% confidence intervals (CI). Patient hospitalization rates were also compared using a GEE logistic regression model and an offset. If multiple comparisons were made within a single model, a closed testing procedure was used to control for type I errors. Changes in the acquisition of patient education parameters were also analyzed using a logistic regression model based on the GEE method. These results were presented as odds ratios and 95% CIs. The duration of exposure during the first year of follow-up was incorporated into the models as an offset, with the models being adjusted based on the endpoint before enrollment. The analyses at 2 years were conducted in the same manner, using the period of exposure during the second year of follow-up, and adjusting the model based on the endpoint after the first follow-up period. A sensitivity analysis was conducted on the primary endpoint “number of days hospitalized for HF,” with patients lost to follow-up being attributed a number of days of hospitalization for the entire year in proportion to the period observed. A *p* value < 0.05 was considered statistically significant. The analyses were conducted using the *R software* environment, version 3.6.0, *R Core Team* (2019) (https://www.R-project.org/). The endpoints were analyzed by comparing the three study periods, namely during the year preceding enrollment, during the first year after enrollment (at 1 year), and during the second year after enrollment (at 2 years).

### 2.8. Administrative and Regulatory Aspects

Informed consent was sought from patients at the initial consultation and kept in the patients’ records. An advisory opinion was obtained from the body in charge of clinical studies at the *Fondation de la Maison du Diaconat de Mulhouse*. The Alsace Regional Health Agency supported the USICAR project (code 67-0021), approved the study as its regulatory aspects in December 2015.

## 3. Results

Over the two-year period between January 2016 and December 2017, 159 adult patients with CHF were enrolled in our study, of whom 117 were men and 42 women. Of those 159 patients, 99 (62.3%) were followed up for two years. Of these, 28 (17.6%) were lost to follow-up during the first year, as were 14 (14.1%) during the second.

### 3.1. Patient Characteristics

The patients had a mean age of 72.9 years (34–96). The male/female sex ratio was 2.8. Hypertension was found in 78 patients (49%), diabetes mellitus in 57 (35.9%), dyslipidemia in 81 patients (50.9), and active smoking was observed in 39 subjects (24.5%). Overweight and obesity was reported in 62 patients (29%). A cardiovascular heredity (myocardial infarction and stroke) was noted in 36 patients (22.6%). Most of the patients had class I–II HF according to the New York Heart Association (NYHA) classification. These class I–II patients accounted for 88.7% of the study population at enrollment (*n* = 141). None of our patients had NYHA class IV HF. In total, 110 patients (62.9%) had reduced left ventricular ejection fraction (<50%). Mean brain natriuretic peptide at enrollment was 519 pg/mL (6–3277). Ischemic heart disease was the most prevalent type of CHF in our population, affecting 81 patients (50.9%). Table 1 summarizes the characteristics of the patients.

### 3.2. Results for Primary and Secondary Endpoints

Table 2 presents overall results for the entire study population during the study periods: The year preceding enrollment, one year, and two years after it. The total number of days that patients were hospitalized for HF was 1343 during the year preceding enrollment, 368 during the first year of follow-up, and 219 during the second.

#### 3.2.1. Primary Endpoint

**Figure 2:**
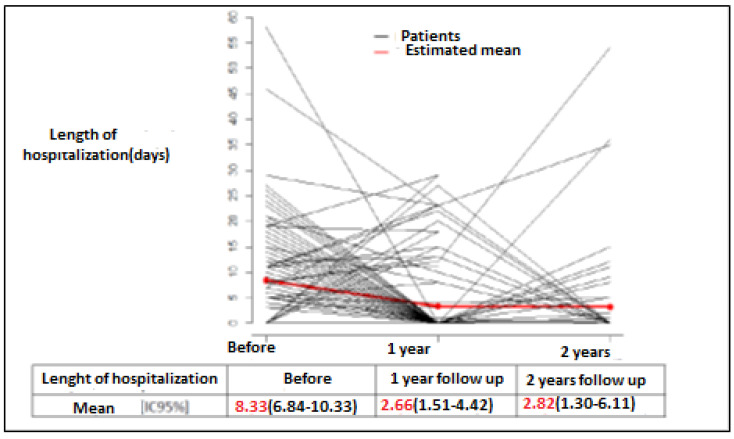
Number of days hospitalized per patient per year before and after inclusion in the USICAR (*Unité de Suivi des Patients Insuffisants Cardiaques*) program. This figure shows the results of the primary endpoint analysis, i.e., the number of days hospitalized for heart failure per patient per follow-up period. The red curve corresponds to the mean number of days hospitalized for heart failure for all patients and for each follow-up period; the curve decreases in the first year, and then remains stable after two years of follow-up.

Figure 2 presents our results for the primary endpoint “number of days hospitalized for HF per patient per year”. The mean number of days was 8.33 (6.84–10.13) during the year preceding enrollment, 2.6 (1.51–4.47) during the first year of follow-up, and 2.82 (1.30–6.11) during the second. As seen in Table 3, this difference was significant when the year preceding enrollment was compared against the first and second years after enrollment (*p* < 0.001 for the two comparisons).

#### 3.2.2. Secondary Endpoints

“Number of days hospitalized for a heart condition other than HF” and “number of hospital stays for HF per patient” are shown in Table 2 before enrollment and during the first and second years of follow-up in the USICAR program. Our findings were, respectively, 274 days, 259 days, and 112 days, and 140 stays, 28 stays, and 20 stays, depending on the study period. When the first of these endpoints was calculated per patient, the number of days of hospitalization for a heart condition other than HF was 1.73 (1.16–2.6), 1.81 (1.04–3.16), and 1.32 (0.57–3.08). The difference was not significant between the study periods (data not shown). The proportion of hospitalizations for HF per patient was 69.5% (60.2–77. 4) preceding enrollment, 16.2% (10–25.2) during the first year of follow-up, and 19.3% (11–31.8) during the second. The difference between before and after enrollment was significant: “1 year/before”: 0.09 [0.04–0.16] (*p* < 0.001) and “2 years/ before”: 0.11 [0.05–0.22] (*p* < 0.001).

Analysis of the treatment in terms of drugs and doses with respect to the standard treatment of CHF patients has showed an improvement. An additional 16% of patients received at least a triple therapy (e.g., angiotensin converting enzyme inhibitors or angiotensin receptor blockers, diuretics, beta-blockers, mineralocorticoid antagonists). Moreover, an additional 22% of patients had their dose optimized (particularly angiotensin converting enzyme inhibitors, angiotensin receptor blockers, and beta-blockers).

Table 4 shows our findings regarding our “patients’ knowledge of their disease” across the three study periods. Their knowledge of all parameters significantly improved at one year (all *p* < 0.001), as well as of all but two parameters at two years (good compliance with low-salt diet, engages in physical activity; *p* = 0.272 and *p* = 0.379, respectively).

Mortality did not differ, with 11 deaths (8.8%) observed at one year and 10 (11.8%) at two years. As to the cost of hospitalizing patients with HF, this was estimated at €658,000 per patient before enrollment in the USICAR program and at €131,000 after enrollment.

During the first and second year, 43 from the 159 patients (27%) and 26 patients from the 99 (26.3%) have benefited from the remote monitoring solution, respectively. All these patients were able to use the solution themselves. The mean “number of days hospitalized for HF per patient per year” was 8.33 during the year preceding enrollment, 2.3 during the first year of follow-up, and 1.7 during the second. This difference was significant when the year preceding enrollment was compared against the first and second years after enrollment *(p* < 0.001 for the two comparisons). The “number of days hospitalized for a heart condition other than HF” was reduced in the group of patient’s beneficiating from the remote monitoring solution during the first and second year of follow-up: 181 and 36 days (versus 259 and 112 in the group without telemedicine) (*p* < 0.01). Table 5 summarizes the means “number of days hospitalized for HF per patient per year” and “number of days hospitalized for a heart condition other than HF” for this subgroup of patients with remote monitoring system.

## 4. Discussion

Our study shows that a program with protocolized follow-up and therapeutic education improved the management of patients with HF, since the mean number of days patients were hospitalized for HF significantly fell from 8.33 during the year preceding enrollment to 2.6 at 1 year after enrollment (*p* < 0.001) (Table 3). Our findings at two years confirm the effectiveness of this systematized follow-up and long-term patient education program. Not only did the number of days of hospitalization for HF significantly decrease, but so too did the frequency of hospitalizations for HF, from 69.5% to 19.3% during the second year of follow-up (*p* < 0.001). A parallel improvement was noted in the patients’ knowledge of their disease, treatments, and dietary and lifestyle guidelines. The USICAR follow-up program did not appear to influence the number of days patients were hospitalized for a heart condition other than HF, since the figures for this parameter remained stable at one year of follow-up. This finding would seem logical given that the present program only applied to HF. Scheduled hospitalizations were more influenced by a patient’s initial type of disease (e.g., arrhythmia and pacemaker) than by the type of therapeutic follow-up they received. This was particularly true at the beginning of the patient’s management. However, owing to the methodology that we employed, it is not known to what extent our findings were attributable to patient education or treatment adjustments, which may have been seen particularly in patients hospitalized for decompensation and during medical follow-up. An additional 16% of patients received at least a triple therapy (e.g., angiotensin converting enzyme inhibitors or angiotensin receptor blockers, diuretics, beta-blockers, mineralocorticoid antagonists). Moreover, an additional 22% of patients had their treatment dose optimized (particularly angiotensin converting enzyme inhibitors, angiotensin receptor blockers, and beta-blockers). In the present experimentation, a significant improvement was also observed with the remote monitoring solution, in the subgroup of patients able to use it (*n* = 26, two years of follow-up).

The results obtained with this program of protocolized follow-up, therapeutic optimization, and education and for more than a quarter of patients with a telemedicine platform are of particular interest given the high hospitalization and re-hospitalization rates among patients with HF reported in the literature, even in NYHA class II–III patients. HF is the main cause of hospitalization among patients aged 65 and over [1,2]. In 2014, 165,093 patients were hospitalized in France for a primary diagnosis of HF. These figures have remained stable in recent years despite the advances that have been made, especially in treatment. The risk of readmission after an initial hospitalization for decompensation is considerable, being between 40% and 60% during the first year in NYHA class III–IV HF according to previous research [7]. In a French study in 152,601 patients who were followed after an initial hospitalization for decompensation in 2009, 25% were readmitted within 30 days for a subsequent episode of decompensation, and 50% were within 12 months [8].

Our study population was noticeably different from the standard French population of patients with CHF [2,6,8]. For instance, the mean age of our patients was 72.9 years versus 78 years in recent French publications, including that of Tupin et al. [6,8]. There was probably also a difference in the physical and mental capacities of our younger patients, who were selected based on their mobility and their comprehension of the USICAR program. This was likely due to the location and manner of patient recruitment, our patients coming directly from community cardiologists or from one of the private clinics in Mulhouse following hospitalization for decompensation. Nevertheless, our patients were representative of the French HF population with regard to type of HF (preserved or reduced ejection fraction) and causes thereof (Table 1). However, it must be noted that 88.7% of the patients enrolled in the present program had moderate HF—that is, NYHA class I–II (Table 1). None of our patients with HF had NYHA class IV disease. In our opinion, this moderate severity brought about by our patient recruitment method may have contributed to the favorable results that we observed in our study, particularly during the two years of the USICAR program. That said, this disease severity does not negate the overall finding of our study, since we used a before-and-after method that meant each patient acted to a certain extent as their own control.

The triggers of decompensation most commonly noted by the French National Authority for Health are poor treatment adherence or excessive fluid retention followed by arrhythmia, myocardial infarction, and, lastly, uncontrolled cardiovascular risk factors such as hypertension [6,7]. Better patient management with improved treatment adherence, diet, and lifestyle seems important if we are to reduce the risk of hospitalization and resulting complications. A 2003 study showed that more than 50% of patients hospitalized for HF displayed signs of exacerbation 15 days before admission [9] and that the early signs of decompensation could be observed up to 30 days beforehand. An early intervention during this period to adjust treatment or dietary and lifestyle behaviors may reduce hospitalization rates. We believe that this is where the effectiveness of the USICAR program probably lies, with a regular patient follow-up and a personalized patient education program being provided by a small team of seasoned practitioners working within a coordinating unit. In this setting, the remote monitoring system may also have a positive impact as previously demonstrated [6,7].

Patient education, optimization of the therapy, and structured follow-up are recognized as key components in informing the patient and getting them actively involved in their own management, thereby improving treatment adherence and promoting compliance with dietary and lifestyle guidelines [5,7,10]. Patients learn to self-monitor so that they can detect tell-tale warning signs of decompensation such as weight gain, dyspnea, edema of the lower limbs, or increased blood pressure and warn healthcare providers to adjust their treatment. In this way, they take ownership of their treatment. Our study showed that the patients improved their knowledge of the disease, treatments, and dietary and lifestyle guidelines through the USICAR program (Table 4). The utility of the program is further enhanced by its forming part of a clinical pathway for patients with CHF, as recommended by the French National Authority for Health [6]. This pathway is run by the USICAR unit in coordination with primary care physicians. Besides the educational aspect of the USICAR program, it is likely that the protocolized follow-up and the use of a remote monitoring platform may have helped optimize HF treatment, although this variable was not studied. Given the follow-up protocol that was set up, it seems probable that patients saw their treatment adjusted during their last hospitalization, with new or missing treatments being added and advice being dispensed on dose escalation and titration. This may have influenced patient outcomes and reduced the re-hospitalization rate. Treatments may also have been adjusted during the observation period. Several studies in the literature have borne this out, illustrating the benefit of adjusting HF treatment [5,7]. This is the case in the present study: +16% of patients received at least a triple therapy and +22% of patients had their treatment dose optimized (particularly angiotensin converting enzyme inhibitors, angiotensin receptor blockers, and beta-blockers).

Various projects and systems have been developed in France to optimize CHF management. One example is the PRADO program launched nationwide by the French national health insurance system [11]. It aims to facilitate the return home and follow-up of patients with HF after they have been hospitalized for decompensation. Meanwhile, healthcare professionals in different hospitals have set up outpatient follow-up programs for patients with HF. These are known as care networks, and examples include the RESICARD HF network in the Greater Paris region and the ICALOR network in Lorraine [12,13,14,15,16]. Each of these networks is organized according to its own principles based on regional resources and circumstances. Like USICAR, the shared goal of these networks is to better coordinate the care of patients with HF by facilitating communication between those involved in their management. Their goal is also to provide patient education. In this context, ICALOR is the only network to have reported similar results to the USICAR program. They saw a 7.19% reduction in hospitalizations for HF compared with the expected number of hospitalizations for the same period (*n* = 1222; 48% and 32% of patients had NYHA class II and III disease) [17]. Telehealth is another promising avenue for optimizing HF patient follow-up. A complete literature review on this topic is provided in [18]. Recently, the randomized prospective *Telemedical Interventional Management in Patients with Heart Failure* (TIM HF 2) study demonstrated the utility of telehealth in 1571 patients [19]. In that study, the all-cause mortality rate was 7.86% (95% CI: 6.14–10.10) per 100 person-years of follow-up in the remote management group versus 11.34% (95% CI: 9.21–13.95) in the usual care group (hazard ratio = 0.70 [95% CI: 0.50–0.96]; *p* = 0.0280). Considering these results and ours, our team has systematically added remote monitoring of all patients with HF to its existing services.

We were unable to draw any conclusions regarding mortality, since the death rate was low and did not differ, with 11 deaths (8.8%) being observed at one year versus 10 (11.8%) at two years. We believe that these findings are consistent with the profile of the patients followed in the present study—no NYHA class IV patients—as well as with data in the literature [1,17,20]. In the report by the French public health surveillance institute, the mortality rate among French patients with HF fell by around 36% between 2000 and 2013 [1]. In men aged over 65, it fell from 952 deaths per 100,000 to 618, while in women in the same age group, it fell from 644 deaths per 100,000 to 409 [1]. Regarding the cost of hospitalizing patients with HF, it was estimated at €658,000 before enrollment in the USICAR program and at €131,000 after enrollment. This represented a saving of more than €500,000. In 2016, the French national health insurance devoted 0.9% of its spending (€1.447 billion) to acute HF [2], 77% of which was allocated to hospital expenses, a figure that amounts to €1.097 billion over one year.

Our study has some limitations, including its retrospective, single-center nature, relatively small sample size, and predominance of NYHA class I and II patients (88.7%). Our study cannot be used to draw any conclusions about patients with severe HF. It also should not be used to draw any conclusions about how long this follow-up of patients with HF should last, although it is tempting to suggest that the USICAR program be pursued long-term. Additionally, the rate of patients lost to follow-up is not insignificant and also weakens our findings. Furthermore, our results are based on a patient education protocol and a mode of organization specific to our team which accommodate available staff resources and local organizational constraints. Moreover, we believe that not all patients who could have, have beneficiated from the telemedicine solution. It is worth emphasizing that these limitations apply to most of the studies in the literature on this topic. That said, our protocol carefully followed the guidelines of the French National Authority for Health and various French and European cardiology societies. We know of no specific protocol that has been approved or supported by any of these professional bodies. Likewise, no studies have been published comparing different protocols or modes of organization. Therefore, the results that we obtained primarily reflect the choices made by the USICAR team. Still, our study does have the merit of documenting the outcome of patients with class I and II HF. Hopefully this will encourage practitioners to conduct a prospective, truly comparative study—protocol versus usual care—on a larger scale with a larger sample size and multiple study centers.

## 5. Conclusions

Despite the limitations cited above, our study is one of the first in France to demonstrate the utility of a protocolized follow-up combined with a patient education program, therapeutic optimization, and a remote monitoring solution in improving the outpatient management of patients with HF, particularly of those with moderate HF. In our opinion, it particularly illustrates the role of patient education in the management of patients with HF [21]. It also has the merit of documenting the outcome of patients with class I and II HF. Our study should encourage practitioners to conduct a prospective, truly comparative study on a larger scale to investigate a regular, systematized follow-up, therapeutic education, and systemic use of telemedicine solution. The positive results of the USICAR program pave the way for its use in telehealth for HF, an approach that is in fact being implemented on a national level in France as part of the experimental ETAPE telehealth program [22].

It must be emphasized that the USICAR program is perfectly in keeping with the medicine of today and, more crucially, with the medicine of tomorrow, that which is personalized, preventive, predictive, participatory, and evidence based. In this setting, artificial intelligence may be of interest.

## Figures and Tables

**Table 1:** Characteristics of the patients included in the USICAR (*Unité de Suivi des Patients Insuffisants Cardiaques*) program.

NYHA class:	
- Class I - Class II - Class III	*n* = 59 (37.1%) *n* = 82 (51.6%) *n* = 18 (11.3%)
Type of heart failure:	
- LVEF < 40% - 50% < LVEF ≥ 40% - LVEF ≥ 50%	*n* = 80 (50.3%) *n* = 30 (18.9%) *n* = 49 (30.8%)
Mean BNP (range)	519 pg/mL (6–3277)
Type of heart disease:	
- Ischemic - Hypokinetic dilated - Rhythmic - Hypertensive - Valvular - Takotsubo	*n* = 81 (50.9%) *n* = 43 (27.1%) *n* = 16 (10.1%) *n* = 10 (6.3%) *n* = 8 (5%) *n* = 1 (0.6%)

NYHA, New York Heart Association; LVEF, left ventricular ejection fraction; BNP, brain natriuretic peptide.

**Table 2:** Overall results in the whole population before and after inclusion in the USICAR (*Unité de Suivi des Patients Insuffisants Cardiaques*) program.

Time Period	TOTAL in the Year Preceding Enrollment	TOTAL at 1 Year of Follow-Up	TOTAL at 2 Years of Follow-Up
Number of patients	159	159	99
Number of days hospitalized for HF	1343	368	219
Number of patients hospitalized for HF	112	23	15
Number of days hospitalized for a heart condition other than HF	274	259	112
Number of deaths	-	11	10
Number of lost to follow-up patients	-	28	14
Number of patients included for < 2 years and being followed up	-	21	-

HF, heart failure.

**Table 3:** Comparison of the ratios of means for the number of days hospitalized for heart failure per patient per year before and after inclusion in the USICAR (*Unité de Suivi des Patients Insuffisants Cardiaques*) program.

	Ratios of Means [95% CI]	Adjusted *p*-Value
1 year/before	0.31 [0.18–0.54]	<0.001
2 years/before	0.34 [0.16–0.73]	<0.001
2 years/1 year	1.08 [0.47–2.48]	0.818

**Table 4:** Data pertaining to the knowledge of patients about heart failure before and after inclusion in the USICAR (*Unité de Suivi des Patients Insuffisants Cardiaques*) program.

Time Period	Already Acquired at the Time of Inclusion	At 1 Year of Follow-Up	At 2 Years of Follow-Up
Weighs himself/herself regularly	45%	85%	89%
Describes the clinical signs of HF	23%	79%	86%
Knows the warning signs	16%	78%	83%
Good treatment compliance	82%	94%	96%
Good knowledge of his/her treatment	39%	79%	83%
Knows which foods are salty	27%	82%	86%
Good compliance with low-salt diet	16%	62%	70%
Is able to quantify his/her water intake	31%	80%	85%
Compliance with water restriction	36%	77%	82%
Engages in physical activity	27%	55%	60%

HF, heart failure.

**Table 5:** Comparison of the means “number of days hospitalized for HF per patient per year” and “number of days hospitalized for a heart condition other than HF” for all patients and for the subgroup of patients with remote monitoring system.

	For All the Patients	For the Subgroup of Patients with the Remote Monitoring System
Number of Days Hospitalized for HF Per patient per year:		
- during the year preceding enrollment - during the first year of follow-up - during the second year of follow-up	8.33 2.6 2.82	8.33 2.3 1.7
Number of days hospitalized for of days hospitalized for a heart condition other than HF:		
- during the year preceding enrollment - during the first year of follow-up - during the second year of follow-up	274 259 112	274 181 36

HF, heart failure. During the first and second year, 43 from 159 patients (27%) and 26 patients from 99 (26.3%) have benefited from the remote monitoring solution, respectively.

## Abbreviations

CHF	chronic heart failure
USICAR	*Unité de Suivi des Patients Insuffisants Cardiaques*
HF	heart failure
ESC	*European Society of Cardiology*
NYHA	New York Heart Association
TIM HF 2	*Telemedical Interventional Management in Patients with Heart Failure*
